# Temporal Trends (from 2008 to 2017) in Functional Limitations and Limitations in Activities of Daily Living: Findings from a Nationally Representative Sample of 5.4 Million Older Americans

**DOI:** 10.3390/ijerph20032665

**Published:** 2023-02-02

**Authors:** Esme Fuller-Thomson, Jason Ferreirinha, Katherine Marie Ahlin

**Affiliations:** 1 Institute for Life Course & Aging, University of Toronto, Toronto, ON M5S 1V4, Canada; 2 Factor-Inwentash Faculty of Social Work, University of Toronto, Toronto, ON M5S 1V4, Canada

**Keywords:** disabilities, education, activities of daily living, functional limitations

## Abstract

This study’s objectives are as follows: (1) to identify the temporal trends in the prevalence and the odds of activities of daily living (ADL) limitations and functional limitations (FLs) among Americans aged 65 and older; (2) to explore if these trends vary by gender and age cohort; (3) to determine if generational differences in educational attainment play a role in the observed temporal trends. A secondary analysis of the American Community Survey (ACS) was conducted for ten consecutive waves of the annual cross-sectional survey (2008–2017). The respondents were community-dwelling and institutionalized adults aged 65 and older (n = 5.4 million). The question on ADLs was “Does this person have difficulty dressing or bathing?”. The question on FLs was “Does this person have serious difficulty walking or climbing stairs?”. There was a substantial decline over the decade in the prevalence of ADL limitations, from 12.1% to 9.6%, and FLs, from 27.3% to 23.5%. If the 2017 prevalence rates had remained at the same level as the 2008 prevalence rates, there would have been an additional 1.27 million older Americans with ADL limitations and 1.89 million with FLs. Adjusting for educational attainment substantially attenuated the odds of the decline for both ADL limitations and FLs.

## 1. Introduction

The limitations in activities of daily living (ADLs), such as bathing and dressing, and functional limitations (FLs), such as problems walking and climbing, are integral components of achieving and maintaining independence in later life [1,2,3]. Approximately one-third (35.2%) of United States (US) adults aged 65 and over report some form of disability [4], with mobility challenges, such as having difficulties with walking and climbing, being the most reported disability among older Americans [5]. Among older adults, FLs and limitations in ADLs are associated with a wide range of negative outcomes, including poor sleep [6], decreased quality of life [7], increased social isolation [8], depressive symptoms [9,10], and pain [11]. Additionally, individuals aged 65 and over living with a disability are more likely to live in poverty [4,12].

The older adult population in the US is projected to grow from 49.2 million in 2016 to 73.1 million by 2030 [13]. Limitations in ADLs are associated with higher Medicare costs after acute hospital admissions, with individuals reporting two or more ADL limitations costing 77% more than those with no functional impairments [14]. Because the prevalence of ADL limitations and FLs increases with age [15], the growth of the American aging population may be accompanied by an increase in the health care expenditures associated with disabilities.

However, if the prevalence of ADL limitations and FLs among older adults declines, the overall increase in the number of disabled older adults may be somewhat attenuated, despite the expansion in the population of older adults. Over the last two decades of the 20th century, there was a decline in the prevalence of ADL limitations in the American population, with a greater decline occurring in the 1990s compared to the 1980s [16]. A systematic review found consistent declines of 1–2.5% per year in the mid- to late-1990s for the prevalence of FLs and ADL limitations in older Americans [17,18]. A more recent publication found improvements in the disability-free life expectancy in the US for both community and institutionalized populations between the years of 1970 and 2010, with an expansion in the number of years that an American could expect to live free of disability after the age of 65 of 2.7 years for men and 2.4 years for women [19]. However, data collected from the 2000–2005 American Community Surveys (ACSs) and the 2004 National Nursing Home Survey (NNHS) suggested an end to the decline in ADL limitations and FLs (Fuller-Thomson et al., 2009).

Based on the projected burgeoning of the older adult population, it is important to determine if the previously identified declines in the prevalence of disability have continued in more recent years. Valid nationally representative data on the trajectory of disabilities in the US are essential to guide planning for health and social care. It is also important to understand the degree to which generational differences in high school and university graduation rates are influencing the observed trajectories. This is necessary so demographic projections of the future burden of disability for those currently in middle age can adequately take into account the potential impact of each birth cohort’s level of educational attainment.

The objectives of this study were to use ten cross-sectional waves (2008–2017) of a nationally representative annual dataset of community-dwelling and institutionalized older Americans, which are as follows:

1. To identify the temporal trends in the prevalence and the odds of activities of daily living (ADL) limitations and functional limitations (FLs) among Americans aged 65 and older;
2. To explore if these trends vary by gender and age cohort;
3. To determine if generational differences in educational attainment play a role in the observed temporal trends.

## 2. Methods

### 2.1. Sample

As has been described elsewhere [20,21,22], this study used ten waves of data from the American Community Surveys (ACSs), which was conducted annually during the decade 2008–2017. The ACS is an annual cross-sectional survey that replaces the long form of the decennial census. The ACS uses a nationally representative sample of Americans living in communities and group quarters (e.g., long-term care facilities). A systematic sample representing each county or county equivalent was selected each month to produce the annual sample [23]. The ACS surveys were conducted by the US Census by mail, the internet, phone, and in-person meetings. Proxy reports were allowed for those living in group quarters [24]. Between 2008–2017, the response rates ranged from 89.9–98.0% in housing units and 94.7–98% in group quarters [25].

For the current study, the sample was restricted to those aged 65 and older available in the public use data sets of the ACS. The total sample size was 5,405,135 respondents. Approximately half a million individuals were included each year, ranging from 467,736 in the 2008 ACS to 610,327 in the 2017 ACS. An Institutional Review Board (IRB) approval was not needed for the secondary analyses of the ACS data as only public-use, non-identifiable data were used.

### 2.2. Measures

*Outcomes of Interest*: For all 10 years of data collection in the ACS, the question on **ADL**s was “Does this person have difficulty dressing or bathing?” and the question on **FLs** was “Does this person have serious difficulty walking or climbing stairs?”. The responses to each question were “yes” or “no”.

*Exposure of Interest*: **The years of data collection** (2008–2017) were coded based on the ACS survey, where 2008 was coded as 1 and 2017 was coded as 10.

*Other variables:***Age** was entered categorically by year of age with a top-coding of 97 into the logistic regression analyses. For the prevalence tables, age was presented in 10-year groups (i.e., 65–74, 75–84, and 85+) and for all respondents aged 65 and older. **Race/ethnicity** was categorized into Hispanic (of any race), Non-Hispanic Whites, Non-Hispanic Blacks, Non-Hispanic American Indian/Alaskan Native, Non-Hispanic Asian-American, Native Hawaiian Pacific Islander, and Non-Hispanic respondents of two or more races.

**Sex** was based on self-reports or proxy reports of male or female. **Education** was categorized as follows: no schooling, some schooling but less than grade 3 completed, each year of education completed from grade 3–11, grade 12 but no diploma, grade 12 regular high school diploma, General Education Diploma, some college but less than 1 year, 1 or more years of college but no diploma, Associate’s degree, bachelor’s degree, master’s degree, professional degree, and doctorate.

### 2.3. Statistical Analysis

Prevalence data for each year of data collection (e.g., 2017) were generated for each sex, and for both sexes combined, for each age cohort (i.e., 65–74, 75–84, and 85+) and for the total sample aged 65 and older. Logistic regression analyses were conducted for the population aged 65 and older for both sexes combined, with the years of data collection being the key variable of interest, and limitations in ADL being the outcome. In the first model, only the years of data collection (where 1 to 10 correspond to 2008 to 2017; entered continuously) were included. In the second model, age (by year of age; entered as categorical variables), race/ethnicity, and sex were added to model 1. Age was entered categorically as opposed to continuously due to the non-linear relationship between age and both ADL limitations and FL. In model 3, the highest level of education completed was entered as a categorical variable in addition to the model 2 variables. The above analyses were repeated for each sex separately. Similar logistic regression analyses were conducted for each of the 3 other age categories (i.e., 65–74, 75–84, and 85+). The above analyses were repeated with FL as the outcome. The odds ratios were calculated per decade rather than per year. All percents and odds ratios were weighted to adjust for non-response and differential selection probabilities [26] so that the findings are representative of the US older adult population. All sample sizes are provided in their unweighted form. All analyses were conducted using IBM SPSS 25.

## 3. Results

The prevalence of limitations in activities of daily living among Americans aged 65 and over declined from 12.1% in 2008 to 9.6% in 2017 (*p* < 0.001) (see Table 1). In the ACS, the question about ADLs was “Does this person have difficulty dressing or bathing?”. As shown in Table 2, column 1, this was the equivalent of a 22% decline in the odds of ADL limitations over the decade (OR = 0.78; 95% CI = 0.77, 0.79). Some of the decline was due to differences in the age structure of the older adult population; when age, race, and sex were taken into account, there was an 18% decline over the decade (OR = 0.82; 95% CI = 0.81, 0.83; see Table 2, column 2). Further adjustments for education substantially attenuated the association such that there was only a 10% decline over the decade when statistical adjustments were performed for education, age, race, and sex (OR = 0.90; 95% CI = 0.89, 0.91; see Table 2, column 3).

The percent decline per decade in the age–sex–race adjusted odds of ADL limitations was 18% when all of the respondents aged 65 and older were included (OR = 0.82; 95% CI = 0.81, 0.83; see Table 2, column 2). This percentage was similar for those aged 85+ (a 19% decline) and those aged 75 to 84 (a 20% decline), but the decline was more modest for those aged 65 to 74 (a 15% decline). Further adjustments for education played a much greater attenuating role for the 65- to 74-year-old cohort, with only a 1% decline over the decade in the odds of ADL remaining after the statistical adjustment, which was not statistically significant, (OR = 0.99; 95% CI = 0.98, 1.01; See Table 2, column 3). In contrast, after additional adjustments for education, those aged 75 to 84 had a 12% decline and those aged 85 and over had a 14% decline.

As shown in Table 3, there was also a substantial and significant (*p* < 0.001) decline in the prevalence of FLs among Americans. In the ACS, the FLs were defined as serious difficulty walking or climbing stairs. The prevalence of FLs among all of those aged 65 and over was 27.3% in 2008, and this declined to 23.5% by 2017.

After adjusting for sex, race, and age, there was a 13% decline in the FLs among older adults over the decade (OR = 0.87; 95% CI = 0.86, 0.88; see Table 4, column 2). When the older adult population was divided into three age groups, the sex–age–race adjusted odds for the FLs varied slightly, with those aged 85 and older having a 12% decline over the decade, while those aged 75 to 84 had a 15% decline, and those aged 65 to 74 had an 11% decline.

Further adjustments for education substantially decreased the decline in the odds of FLs to only 4% per decade for the total older adult population aged 65 and older (OR = 0.96; 95% CI = 0.95, 0.97; see Table 4, column 3), which reflected a 2% increase in the odds of FLs for those aged 65 to 74 and a 7% and an 8% decline over the decade for those aged 75 to 84, and 85 and over, respectively.

If the prevalence of both disabilities had remained at the 2008 levels across the decade, there would have been an additional 1,274,000 Americans with ADL limitations in 2017 and an additional 1,890,000 Americans with FLs.

## 4. Discussion

The functional health of older Americans has improved, as measured by declines in the prevalence of ADL limitations and FLs from 2008 to 2017. During this period, the prevalence of reported disabilities among older Americans aged 65 and older decreased from 12.1% to 9.6% for ADL limitations and from 27.3% to 23.5% for FLs. When adjustments were made for sex, race, and age, there was a decline in the odds of ADL limitations and FLs of 18% and 13%, respectively, over the decade for the combined sample of those aged 65 and older. The decline in the prevalence of disabilities was substantial; if the prevalence of limitations in ADL and FLs had remained constant across the decade at the 2008 prevalence, there would have been an additional 1.27 million older Americans with ADL limitations and 1.89 million older Americans with FLs in 2017.

**The Key Role of Educational Attainment:** Analyses indicated that education played a significant role in attenuating the odds of the decline for both ADL limitations and FLs across the age groups. Of particular note, among the 65- to 74-year-old age group, including education in the analysis nearly eliminated the decline in the odds of ADL limitations to only 0.5% across the decade. Similarly, taking into account the highest level of education achieved decreased the decline in the odds of FLs from 13% to 4% for adults aged 65 and older and, interestingly, resulted in a 2% *increase* over the decade in the odds of FLs for men. Several other studies have suggested that higher educational attainment may explain the observed decline in the prevalence of ADL limitations and FLs [27,28,29,30].

There has been an increasing trend in higher educational attainment in the American population, with the proportion of adults aged 65 years and over with a high school diploma increasing from 72.0% in 2003 to 84% in 2015, and those with a bachelor’s degree increasing from 17.0% to 27% during the same time period [31]. There are several reasons why education may decrease the likelihood of the development of ADL limitations and FLs later in life. Greater educational attainment is correlated with higher health literacy, and, in turn, higher health literacy is associated with an increased level of participation in health-promoting behaviors and lower levels of disability [32]. Additionally, lower educational attainment is associated with smoking [33] and cardiovascular conditions, such as hypertension, stroke, and diabetes [34,35]. These factors are known predictors for the development of later-life disabilities [36,37]. Older adults with less education are also more likely to have had a career involving physically demanding work, to have retired due to disabilities resulting from back pain [38,39], and to have mobility loss [30].

**Beyond Education—Other Possible Factors that may be Influencing the Decline in Disabilities:** In addition to education, decreasing trends in other risk factors, such as smoking, may be contributing to the decline in ADL limitations and FLs over the decade. American smoking rates have substantially declined since the 1960s [40]. A systematic review showed that current or former smoking is a risk factor for the development of functional limitations in older adults [36], and a nationally representative study in Sweden showed that heavy smokers had higher levels of mobility impairment compared to non-smokers [41]. Additionally, eliminating smoking has been found to extend life and increase the number of years lived without disabilities [42,43].

Stroke is another risk factor that has shown marked improvements over time. Stroke incidence has declined by 42% between the 1970s and 2000s [44]. Stroke is the third leading cause of long-term disabilities worldwide, and almost 50% of stroke survivors experience limitations in ADLs [45,46].

Environmental factors such as the decreasing levels of air pollutants may also be impacting the decline in ADL limitations and FLs. Common air pollutants such as carbon monoxide, nitrogen dioxide, and fine particulate matter (PM_2.5_) have declined by 65%, 51%, and 43%, respectively, between 2000 and 2019 [47]. The exposure to higher levels of air pollutants is associated with an increased risk of developing chronic health conditions, such as cardiovascular disease, stroke, and diabetes [48]. Given that chronic health conditions influence the progression of physical disabilities in older adults [36], lower levels of air pollutants may be contributing to fewer chronic diseases and declines in disabilities among older Americans.

An additional environmental factor to consider is the phaseout of leaded gasoline in the United States, which began in the 1970s. Lead is a known neurotoxin, and high levels of lead exposure are associated with declines in cognition [49]. The phaseout of leaded gasoline substantially decreased the blood and bone lead levels in the 1970s, 1980s, and 1990s [50]. Dementia is a condition with a long latency period of many decades. It has been hypothesized that the steep decline in cognitive impairment among older Americans may be linked to the decline in lead-related air pollution many decades earlier [22]. Cognitive decline is associated with an increased incidence of mobility impairments and mobility declines, with lower levels of global cognition relating to more rapid mobility declines [51].

Additionally, decreasing incidence of hip fracture may be a factor in the observed decline in ADL limitations and FLs, as studies have shown that hip fracture survivors have significantly worse mobility and functional independence compared to those who have not experienced such injuries [52]. There has been a decrease in the annual incidence of hip fractures in the US beginning in 1995 [53,54], with an analysis of US Medicare claims from 2002 to 2015 revealing that there was a decline in hip fracture rates each year from 2002 to 2012 before plateauing from 2013 to 2015 [55].

Obesity is associated with greater difficulties in mobility activities, such as walking, stair climbing, and chair lifting [56], and it is a risk factor for the development of ADL limitations [57]. The Baby Boomer generation (those born between 1946 and 1965) has significantly higher rates of obesity compared to preceding generations [58]. Furthermore, on average, Baby Boomers became obese at an earlier age than earlier generations [59]. The current study found that the majority of the improvement in disabilities was evident in those aged 75 and older, with much more modest improvements in the Baby Boom subsample of those aged 65–74. These findings are in keeping with the above-discussed obesity trends in the different age cohorts. It may be that the observed trajectory of disability improvements from 2008 to 2017 will stall or reverse in future decades as the Baby Boom generation, with its related obesity pandemic, fills an ever-expanding portion of the older adult population.

### Strengths and Limitations

Several limitations of this study must be considered. The ACS does not include factors that are associated with disability, such as health behaviors (smoking, physical inactivity, and obesity), health conditions (stroke, diabetes, hip fractures, etc.), and lifetime air pollution exposure; thus, examination into the impact of these factors on the observed decline in disability could not be explored. It would be beneficial for future research to collect information on these important factors to understand the extent to which they are driving the observed improvements in the prevalence of disabilities. Additionally, the ACS relies on participant self-reports. It would be better to have objective measurements of the disabilities. A promising new technology has emerged that may make it possible to accurately assess disabilities remotely [60].

Despite these limitations, this study was conducted using a nationally representative dataset of 5.4 million community-dwelling and institutionalized older Americans with high response rates ranging from 89.9% to 98.0% annually. Thus, the current analyses provide one of the largest studies on the trends in disabilities in the US in recent years.

## 5. Conclusions

Across the 10-year period, there were substantial decreases in the age–sex–race adjusted odds of disability among older Americans, with a 20% decline in ADL limitations and a 13.8% decline in FLs. Had the 2008 prevalence of ADL limitations and FLs remained constant across the decade, this percentage decline would translate to 1.27 million and 1.89 million fewer older adults developing ADL limitations and FLs, respectively.

Even with the observed substantial improvements in the disability prevalence, we have to consider that the number of older adults will continue to increase substantially, with the final members of the Baby Boom demographic bulge reaching the age of 65 by 2030. These demographic trends will lead to an increase in the number of adults experiencing disabilities later in life. Because of this, future research needs to continue to monitor the disability trends.

It is also important to implement prevention and treatment strategies as this generation continues to age in the coming decades. Evidence-based strategies to prevent disabilities include the following: fall prevention programs, such as structured exercise programs; the inspection and removal of environmental hazards, such as loose rugs; the provision of environmental improvements, such as bath grab rails and adequate lighting [61]. Among those with ADL limitations or FLs, cognitive interventions to enhance problem solving and resource utilization, physical exercise training for functional movement, and resistance training have been shown to ameliorate the levels of disability [62].

It would be of enormous financial and social benefit to the US if the decline in the prevalence of FLs and ADL limitations among older adults continues unabated in the decades to come.

## Figures and Tables

**Table 1:** Temporal trends in the prevalence of activities of daily living (ADL ^1^) limitations by decade (2008–2017), gender, and age cohort (ages 65–74; 75–84; 85+; full sample aged 65 and older). Source: American Community Surveys Data (n = 5405,135). (The prevalences are weighted; the sample sizes are unweighted and provided in thousands).

Year		Age 65–74			Age 75–84			Age 85+			Age 65+ (Total)		
	Unweighted Sample (in Thousands)	Total	Males	Females	Total	Males	Females	Total	Males	Females	Total	Males	Females
2008	468	5.8	5.3	6.2	13.1	10.8	14.7	33.3	25.9	36.7	12.1	9.3	14.2
2009	480	5.5	5.1	5.8	12.6	10.7	13.9	32.1	24.6	35.5	11.5	8.9	13.5
2010	491	5.3	4.8	5.6	12.0	10.2	13.4	30.7	24.2	33.8	10.9	8.5	12.8
2011	524	5.4	4.9	5.8	12.2	10.2	13.6	30.9	23.7	34.4	11.0	8.5	13.0
2012	538	5.3	4.9	5.6	11.9	10.0	13.3	30.1	24.1	33.2	10.7	8.4	12.4
2013	544	5.2	4.8	5.5	11.6	9.5	13.1	30.4	23.7	33.7	10.5	8.1	12.3
2014	566	5.2	4.9	5.4	11.6	9.9	12.8	30.3	24.3	33.4	10.4	8.3	12.0
2015	583	5.1	4.8	5.3	11.2	9.7	12.4	30.0	23.9	33.2	10.1	8.1	11.7
2016	601	5.1	5.0	5.2	11.1	9.5	12.4	29.8	24.1	32.9	10.0	8.2	11.4
2017	610	4.9	4.7	5.0	10.6	9.0	11.8	29.7	23.9	32.8	9.6	7.8	11.0
Unweighted Totals (×1000)	5405	3004	1404	1600	1681	723	958	718	242	476	5405	2371	3034
Linear by linear association		278	32	293	757	197	514	278	16	221	2703	398	2266
*p*-value		<0.001	<0.001	<0.001	<0.001	<0.001	<0.001	<0.001	<0.001	<0.001	<0.001	<0.001	<0.001

^1^ The question on ADLs was “Does this person have difficulty dressing or bathing?”.

**Table 2:** Temporal trends in the odds of activities of daily living (ADL ^1^) limitations per decade. Odds and 95% confidence intervals (*p* < 0.001, unless otherwise indicated). Source: American Community Surveys annual data from 2008–2017 (n = 5,405,135).

Age Cohort (Sample Size)	Unadjusted Odds of Activities of Daily Living Limitations per Decade (2008–2017) (95% CI)	Odds of Activities of Daily Living Limitations per Decade (2008–2017), Adjusted for Age, Race (Includes Adjustment for Sex in Non-Gender-Specific Analyses) (95% CI)	Odds of Activities of Daily Living Limitations per Decade (2008–2017) (95% CI), Adjustment for Age, Race, (Includes Adjustment for Sex in Non-Gender-Specific Analyses) and Education. (95% CI)
Age 65–74			
Both Genders 65–74 (n = 3,004,467)	0.86 (0.85, 0.88)	0.85 (0.84, 0.87)	0.99 (0.98, 1.01), *p* = 0.50
Men 65–74 (n = 1,404,814)	0.93 (0.90, 0.95)	0.92 (0.89, 0.94)	1.06 (1.03, 1.09)
Female 65–74 (n = 1,599,653)	0.81 (0.80, 0.83)	0.81 (0.79, 0.83)	0.94 (0.92, 0.97)
Age 75–84			
Both Genders 75–84 (n = 1,681,964)	0.80 (0.78, 0.81)	0.80 (0.79,0.82)	0.88 (0.86, 0.89)
Men 75–84 (n = 723,892)	0.82 (0.80, 0.85)	0.82 (0.80, 0.85)	0.90 (0.87, 0.92)
Female 75–84 (n = 958,072)	0.79 (0.77, 0.81)	0.79 (0.77, 0.81)	0.86 (0.84, 0.88)
Age 85 +			
Both Genders 85+ (n = 718,704)	0.86 (0.85, 0.88)	0.81 (0.80, 0.83)	0.86 (0.84, 0.87)
Men 85+ (n = 242,303)	0.94 (0.91, 0.97)	0.88 (0.85, 0.91)	0.92 (0.89, 0.96)
Female 85+ (n = 476,401)	0.85 (0.84, 0.87)	0.79 (0.77, 0.81)	0.83 (0.82, 0.85)
Total (age 65+)			
Both Genders 65+ (n = 5,405,135)	0.78 (0.77, 0.78)	0.82 (0.81, 0.83)	0.90 (0.89, 0.91)
Men 65+ (n = 2,371,009)	0.85 (0.84, 0.86)	0.87 (0.86, 0.89)	0.96 (0.94, 0.97)
Female 65+ (n = 3,034,126)	0.75 (0.74, 0.76)	0.80 (0.79, 0.81)	0.87 (0.86, 0.88)

^1^ The question on ADLs was “Does this person have difficulty dressing or bathing?”. *p*-values < 0.01 unless otherwise stated.

**Table 3:** Temporal trends in the prevalence of functional limitations (FLs ^1^) by decade (2008–2017), gender, and age cohort (ages 65–74, 75–84, and 85+; full sample aged 65 and over). Source: American Community Surveys Data (n = 5,405,135). (The prevalences are weighted; the sample sizes are unweighted and provided in thousands).

Year		Age 65–74			Age 75–84			Age 85+			Age 65+ (Total)		
	Unweighted Sample (in Thousands)	Total	Males	Females	Total	Males	Females	Total	Males	Females	Total	Males	Females
2008	468	17.6	15.2	19.7	30.6	25.9	34.0	55.1	46.3	59.1	27.3	21.9	31.2
2009	480	17.3	15.0	19.3	30.0	25.6	33.2	54.0	45.7	57.9	26.6	21.6	30.4
2010	491	16.8	14.6	18.7	29.2	24.8	32.4	52.9	45.4	56.5	25.7	21.0	29.4
2011	524	16.6	14.3	18.6	29.4	24.9	32.6	52.6	44.8	56.4	25.6	20.8	29.3
2012	538	16.3	14.3	18.0	28.7	24.3	31.9	52.1	45.1	55.6	25.0	20.5	28.4
2013	544	16.5	14.7	18.1	28.8	24.4	32.1	52.7	45.7	56.2	25.0	20.7	28.4
2014	566	16.4	14.9	17.8	28.4	24.1	31.7	52.7	45.4	56.5	24.7	20.6	28.0
2015	583	16.3	14.7	17.7	27.8	23.8	30.8	52.4	45.5	56.1	24.3	20.4	27.4
2016	601	16.1	14.4	17.5	27.8	23.9	30.8	52.3	45.6	55.9	24.1	20.2	27.2
2017	610	15.9	14.5	17.1	27.0	23.3	29.8	51.8	45.1	55.5	23.5	20.0	26.4
Unweighted Totals (×1000)	5405	3004	1404	1600	1682	724	958	719	242	476	5405	2371	3034
Linear by linear association		456	19	572	854	206	573	151	1.95	132	3193	389	2836
*p*-value		<0.001	<0.001	<0.001	<0.001	<0.001	<0.001	<0.001	0.16	<0.001	<0.001	<0.001	<0.001

^1^ The question on functional limitations (FLs) was “Does this person have serious difficulty walking or climbing stairs?”.

**Table 4:** Temporal trends in the odds of functional limitations (FLs ^1^) per decade. Odds and 95% confidence intervals (*p* < 0.001, unless otherwise indicated). Source: American Community Surveys annual data 2008–2017 (n = 5,405,135).

Age Cohort (Sample Size)	Unadjusted Odds of Functional Limitations per Decade (2008–2017) (95% CI)	Odds of Functional Limitations per Decade (2008–2017), Adjusted for Year and Age, Race (Includes Adjustment for Sex in Non-Gender-Specific Analyses) (95% CI)	Odds of Functional Limitations per Decade (2008–2017) (95% CI), Adjustment for Year of Age, Race, (Includes Adjustment for Sex in Non-Gender-Specific Analyses), and Education. (95% CI)
Age 65–74			
Both Genders 65–74 (n = 3,004,467)	0.89 (0.88, 0.90)	0.89 (0.88, 0.90)	1.02 (1.01, 1.03)
Men 65–74 (n = 1,404,814)	0.96 (0.95, 0.98)	0.96 (0.94, 0.97)	1.10 (1.08, 1.12)
Female 65–74 (n = 1,599,653)	0.84 (0.83, 0.86)	0.84 (0.83, 0.85)	0.97 (0.95, 0.98)
Age 75–84			
Both Genders 75–84 (n = 1,681,964)	0.84 (0.83, 0.85)	0.85 (0.84, 0.86)	0.93 (0.92, 0.94)
Men 75–84 (n = 723,892)	0.87 (0.86, 0.89)	0.88 (0.86, 0.89)	0.95 (0.93, 0.97)
Female 75–84 (n = 958,072)	0.83 (0.82, 0.85)	0.84 (0.82, 0.85)	0.91 (0.90, 0.92)
Age 85+			
Both Genders 85+ (n = 718,704)	0.90 (0.89, 0.92)	0.88 (0.86, 0.89)	0.92 (0.91, 0.94)
Men 85+ (n = 242,303)	0.98 (0.95, 1.01), *p* = 0.16	0.94 (0.91, 0.97)	0.99 (0.96, 1.02), *p* = 0.38
Female 85+ (n = 476,401)	0.89 (0.87, 0.91)	0.85 (0.83, 0.86)	0.90 (0.88, 0.91)
Total (age 65)			
Both Genders 65+ (n = 5,405,135)	0.82 (0.82, 0.83)	0.87 (0.86, 0.88)	0.96 (0.95, 0.97)
Men 65+ (n = 2,371,009)	0.90 (0.89, 0.91)	0.92 (0.91, 0.94)	1.02 (1.01, 1.03)
Female 65+ (n = 3,034,126)	0.79 (0.78, 0.80)	0.84 (0.83, 0.85)	0.93 (0.92, 0.93)

^1^ The question on functional limitations (FL) was “Does this person have serious difficulty walking or climbing stairs?”. *p*-values < 0.01 unless otherwise stated.

## Data Availability

The US Census Bureau provides these data in public use files at https://www.census.gov/programs-surveys/acs/data.html (accessed on 10 January 2023).
